# The nomogram for the prediction of overall survival after surgery in patients in early‐stage NSCLC based on SEER database and external validation cohort

**DOI:** 10.1002/cam4.6751

**Published:** 2023-12-26

**Authors:** Hao Zhang, Jingtong Zeng, Xianjie Li, Bo Zhang, Hanqing Wang, Quanying Tang, Yifan Zhang, Shihao Bao, Lingling Zu, Xiaohong Xu, Song Xu, Zuoqing Song

**Affiliations:** ^1^ Department of Lung Cancer Surgery Tianjin Medical University General Hospital Tianjin China; ^2^ Tianjin Key Laboratory of Lung Cancer Metastasis and Tumor Microenvironment Lung Cancer Institute, Tianjin Medical University General Hospital Tianjin China; ^3^ Colleges of Nursing Tianjin Medical University Tianjin China

**Keywords:** nomogram, non‐small cell lung cancer, prognostic factor, the Surveillance, Epidemiology, and End Results Program (SEER)

## Abstract

**Background & Aims:**

Currently, there is a lack of effective tools for predicting the prognostic outcome of early‐stage lung cancer after surgery. We aim to create a nomogram model to help clinicians assess the risk of postoperative recurrence or metastasis.

**Materials and Methods:**

This work obtained 16,459 NSCLC patients based on SEER database from 2010 to 2015. In addition, we also enrolled 385 NSCLC patients (2017/01‐2019/06) into external validation cohort at Tianjin Medical University General Hospital. Univariable as well as multivariable Cox regression was carried out for identifying factors independently predicting OS. In addition, we built a nomogram by incorporating the above prognostic factors for the prediction of OS.

**Results:**

Tumor size was positively correlated with the risk of poor differentiation. Advanced age, male and adenocarcinoma patients were factors independently predicting poor prognosis. The risk of white race is higher, followed by Black race, Asians and Indians, which is consistent with previous study. Chemotherapy is negatively related to prognostic outcome in patients of Stage IA NSCLC and positively related to that in those of Stage IB NSCLC. Lymph node dissection can reduce the postoperative mortality of patients. AUCs of the nomograms for 1, 2, and 3‐year OS was 0.705, 0.712, and 0.714 for training cohort, while those were 0.684, 0.688, and 0.688 for validation cohort.

**Conclusions:**

The nomogram could be used as a tool to predict the postoperative prognosis of patients with Stage I non‐small cell lung cancer.

## 1 INTRODUCTION

Lung cancer not only has a very high incidence, but also the first mortality cancer in the word.^1^ Currently, more than 85% of these cases are classified as non‐small cell lung cancer (NSCLC).^2^ and its 5‐year survival rate is expected to be 26% percent that has improved only slightly over the past few decades.^3^

As computed tomography (CT) popularizes, more and more early‐stage NSCLC diagnosed. Nevertheless, 16.79%–31% of all Stage I patients undergoing radical resection experience recurrence or metastasis.^4^, ^5^, ^6^, ^7^, ^8^ Most recurrences or metastasis occurred within the first 2 years after surgery.^9^ Once recurrence or metastasis occurs, the prognosis of patients is very poor. Previous studies have shown that the 2‐year post‐recurrence or distant metastasis survival rates for I stage patients were 15.1%–55.1%.^4^, ^5^, ^6^, ^7^

Therefore, it is necessary to create a tool supporting clinical decision‐making, according to the individual factors for lung cancer patients. The tumor lymphatic metastasis (TNM) classified system released by the American Joint Committee on Cancer (AJCC) and the International Union against Cancer (UICC) has been extensively applied in predicting tumor stage and prognostic outcome in tumor patients.^10^ But TNM classification system can not sufficiently estimate clinical prognosis because numerous clinicopathological factors influence a patient's prognosis. All of these factors are known to affect a cancer patient's prognosis, such as age, sex, race, and resection type.^11^, ^12^, ^13^, ^14^

Nomogram, widely used in medicine and oncology as a statistical tool.^15^, ^16^, ^17^, ^18^ There have been many previous studies applying nomogram to predict the risk of lung cancer, but few of them have focused on Stage I postoperative NSCLC. In addition, this study also included the number of lymph nodes dissected and the number of lymph node stations dissected in the nomogram.^19^, ^20^, ^21^, ^22^, ^23^ We aim to create a nomogram model for postoperative survival using the data form SEER database during 2010–2015. In addition, we also enrolled 385 NSCLC patients into external validation cohort for validating model effectiveness. The nomogram is assessed with receiver operating characteristic (ROC) curve, calibration curve, together with decision curve analysis (DCA) and concordance index (C‐index).

## 2 PATIENTS AND METHODS

### 2.1 Data collection

**FIGURE 1:**
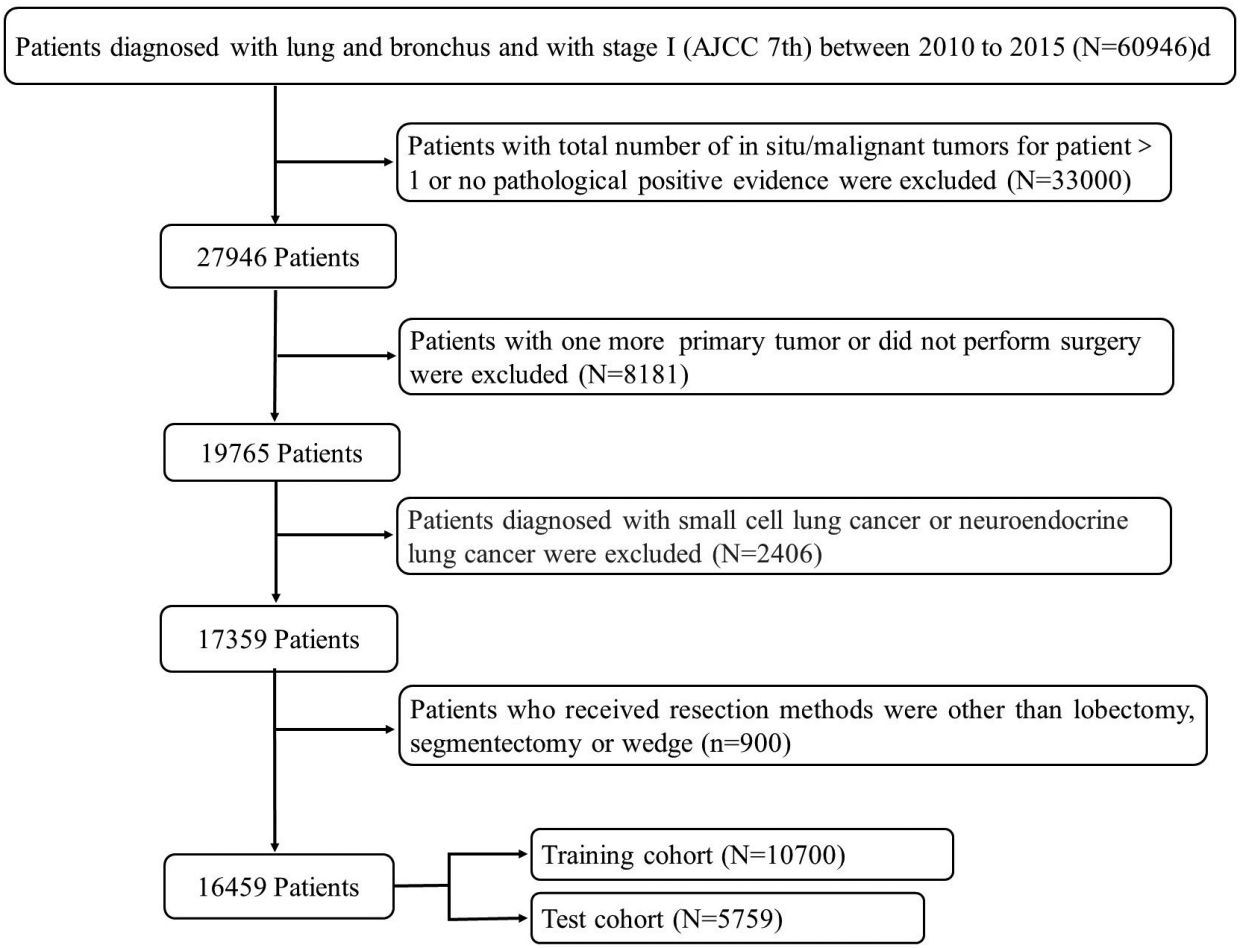
Flow chart for screening NSCLC patients in SEER.

Data were collected with SEER*Stat software (Version 8.4.0.1), which included clinical and demographic data. NSCLC patients were recruited during 2010–2015 in line with relevant eligibility criteria. Patients with incomplete or missing information were excluded. Patients below were included: (1) diagnosis made during 2010–2015 (2); lung cancer being the only malignancy; (3) only one primary tumor; (4) lung cancer confirmed by pathological histological examination; (5) Stage I (AJCC 7th); (6) Surgery was performed and the method of resection were wedge resection, segmentectomy or lobectomy; (7) Oncology histology codes: squamous cell carcinoma (8052, 8070‐8074); adenocarcinoma (8140, 8250‐8255, 8260, 8290, 8310, 8323, 8430, 8480, 8481, 8490, 8550, 8551) adenosquamous carcinoma (8560); large cell lung cancer (8012, 8013) and other NSCLC (8046, 8010, 8050, 8575) (Figure 1).

Moreover, we retrospectively collected Stage I NSCLC patients undergoing surgery from as external validation cohort from 2017.01 to 2019.06 at Tianjin Medical University General Hospital in line with criteria below: (1) Stage I (AJCC 7th); (2) Surgery was performed; (3) lung cancer confirmed by pathological histological examination; (4) only one primary tumor.

Patients below were excluded” (1) those who received preoperative neoadjuvant therapy; (2) those developing other tumors; (3) those refusing follow‐up.

### 2.2 Development of the nomogram and statistical analysis

The categorical variables were represented by numbers (*n*) and percentages (%), and compared by the chi‐square test. Through a no‐replacement random sampling method, all samples were classified as training or validation cohort at the 6.5:3.5 ratio. Training cohort was used to create nomogram, while validation cohort was applied in validating results of training cohort. Factors associated with OS were obtained by univariable Cox regression, which were later incorporated into multivariable regression to identify independent risk factors. Variables with *p <* 0.05 upon univariable Cox regression were incorporated for multivariable Cox regression. We also determined hazard ratios (HR) as well as 95% confidence intervals (CI) for the filter variables. According to multivariable regression, we then created prognosis prediction nomograms for 1‐, 2‐, and 3‐year OS. AUC is defined in the context of ROC curve analysis as the area enclosed by coordinate axes and ROC curve. Nomogram diagnostic significance is represented by AUC, which ranged from 0.5 to 1. The bootstrap method was used to conduct calibration curve analysis using 1000 resamples. Horizontal coordinate represents threshold probability, while vertical coordinate indicates net benefit rate following subtraction of benefits from the harm. If different evaluation approaches reached up to the certain value, the temporal event risk probability was represented by Pi. If Pi reached the particular threshold (Pt), it was positive. The model curve close to two reference lines indicates that this model does not have any value, whereas a curve on the top of reference line within a broad threshold interval indicates that the model is more valuable.

Statistical analysis was conducted with R Statistical Software (version 4.1.3) and IBM SPSS Statistics Software, version 22.0. Clinicopathological features were included to construct the nomogram using “rms”, “foreign”, “survival” and “regplot” packages. The “caret” package was used for randomization and the “survival” package was used for C‐index. Relations between different variables and OS were assessed by Kaplan–Meier survival curve analysis.

## 3 RESULTS

### 3.1 Patient features

This work obtained altogether 16,459 NSCLC patients based on SEER database during 2010–2015. 65% of patients included based on SEER database (10,700 patients) were incorporated into training cohort, while the rest (5759 patients) into internal validation cohort. In addition, 385 NSCLC patients (2017/01‐2019/06) were also included into external validation cohort at Tianjin Medical University General Hospital (referred to as TJMUGH cohort). Median follow‐up time for all 385 surgically resected Stage I NSCLC patients was 45.1 months (mean 43.5 months). In the SEER cohort, the 80% (13,163 patients) were over 60 years old, 56% (9210 patients) were male patients, and 83% (13654) were White, while the rest were Black or other. All variables except gender were not significantly different between training and internal validation groups, possibly as a result of differences in treatment methods according to age and race at randomization. Tables 1 and 2 display more details of patient clinicopathological features.

**TABLE 1:** Basic demographics and clinical features of NSCLC patients of train and test cohort.

	Total *n* = 16,459	Train *n* = 10,700 (65%)	Test *n* = 5759 (35%)	*p*‐Value
Age				
≤59	3296 (20.03%)	2163 (20.22%)	1133 (19.67%)	0.2701
≥80	1790 (10.87%)	1187 (11.09%)	603 (10.47%)	
60–79	11,373 (69.1%)	7350 (68.69%)	4023 (69.86%)	
Gender				
Female	9210 (55.96%)	6087 (56.89%)	3123 (54.23%)	0.0011
Male	7249 (44.04%)	4613 (43.11%)	2636 (45.77%)	
Marital status				
Divorced	2034 (12.36%)	1331 (12.44%)	703 (12.21%)	0.7612
Married	9038 (54.91%)	5861 (54.78%)	3177 (55.16%)	
Single	2146 (13.04%)	1383 (12.92%)	763 (13.25%)	
Widowed	2493 (15.15%)	1624 (15.18%)	869 (15.09%)	
Unknown	748 (4.54%)	501 (4.68%)	247 (4.29%)	
Race				
Asian or Pacific Islander	1324 (8.04%)	880 (8.23%)	444 (7.71%)	0.4144
Black	1422 (8.64%)	903 (8.44%)	519 (9.01%)	
White	13,654 (82.96%)	8877 (82.96%)	4777 (82.95%)	
Unknown	59 (0.36%)	40 (0.37%)	19 (0.33%)	
Histologic type				
Adenocarcinoma	11,626 (70.64%)	7572 (70.77%)	4054 (70.39%)	0.9212
Squamous carcinoma	3895 (23.66%)	2521 (23.56%)	1374 (23.86%)	
Adenosquamous	363 (2.21%)	231 (2.16%)	132 (2.29%)	
Large cell	302 (1.83%)	194 (1.81%)	108 (1.88%)	
NSCLC(NOS)	273 (1.66%)	182 (1.70%)	91 (1.58%)	
Tumor size				
T1a	6710 (40.77%)	4359 (40.74%)	2351 (40.82%)	0.9994
T1b	3596 (21.85%)	2340 (21.87%)	1256 (21.81%)	
T1(NOS)	29 (0.17%)	19 (0.18%)	10 (0.17%)	
T2	6124 (37.21%)	3982 (37.21%)	2142 (37.20%)	
Resection type				
Lobectomy	12,977 (78.84%)	8435 (78.83%)	4542 (78.87%)	0.8656
Segmentectomy	779 (4.73%)	513 (4.80%)	266 (4.62%)	
Wedge	2703 (16.42%)	1752 (16.37%)	951 (16.51%)	
No. of resected LN stations				
0	1497 (9.11%)	941 (8.79%)	556 (9.66%)	0.2778
1–3	2682 (16.29%)	1765 (16.50%)	917 (15.92%)	
≥4	11,551 (70.18%)	7518 (70.26%)	4033 (70.03%)	
Others	729 (4.42%)	476 (4.45%)	253 (4.39%)	
No. of resected LNs				
0	1538 (9.34%)	980 (9.16%)	558 (9.69%)	0.3742
1–4	3758 (22.83%)	2479 (23.17%)	1279 (22.21%)	
5–8	3925 (23.85%)	2574 (24.06%)	1351 (23.46%)	
≥9	6153 (37.38%)	3973 (37.13%)	2180 (37.85%)	
Unknown	1085 (6.59%)	694 (6.48%)	391 (6.79%)	
Chemotherapy				
No	15,339 (93.20%)	9960 (93.08%)	5379 (93.4%)	0.4599
Yes	1120 (6.80%)	740 (6.92%)	380 (6.6%)	
Radiation				
No	15,998 (97.21%)	10,393 (97.13%)	5605 (97.33%)	0.5004
Yes	461 (2.79%)	307 (2.87%)	154 (2.67%)	
Grade				
Well differentiated	3371 (20.48%)	2190 (20.47%)	1181 (20.51%)	0.9322
Moderately differentiated	7558 (45.92%)	4920 (45.98%)	2638 (45.80%)	
Poorly differentiated	4478 (27.21%)	2919 (27.28%)	1559 (27.07%)	
Undifferentiated	164 (1.00%)	106 (0.99%)	58 (1.01%)	
Unknown	888 (5.39%)	565 (5.28%)	323 (5.61%)	

**TABLE 2:** Basic demographics and clinical features of NSCLC patients of TJMUGH cohort.

Total	No. of percentage *n* = 385
Age	
≤59	107 (27.8%)
60–79	271 (70.4%)
≥80	7 (1.8%)
Gender	
Female	193 (50.1%)
Male	192 (49.9%)
Marital status	
Married	359 (93.2%)
Divorced	3 (0.8%)
Single	17 (4.4%)
Widowed	6 (1.6%)
Histologic type	
Adenocarcinoma	327 (84.9%)
Adenosquamous	56 (14.5%)
Squamous carcinoma	2 (0.5%)
Tumor size	
T1a	243 (60.1%)
T1b	90 (23.4%)
T2	52 (13.5%)
Resection type	
Lobectomy	253 (65.7%)
Segmentectomy	34 (8.8%)
Wedge	98 (25.5%)
No. of resected LN stations	
0	44 (11.4%)
1–3	69 (17.9%)
≥4	272 (70.7%)
No. of resected LNs	
0	45 (11.7%)
1–4	41 (10.7%)
5–8	59 (15.3%)
≥9	240 (62.3%)
Chemotherapy	
No	248 (64.4%)
Yes	137 (35.6%)
Grade	
Well differentiated	327 (84.9%)
Moderately differentiated	38 (9.9%)
Poorly differentiated	20 (5.2%)

The patients of SEER database cohort were excluded if they had been followed for less than 2 years and had not died by the time of their next visit. As to the TJMUGH cohort, the inclusion criteria were as follows: (1) Patients receiving preoperative neoadjuvant therapy; (2) Patients with other tumors; (3) Patients who refused follow‐up. Overall survival (OS) was our endpoint, while follow‐up period was the duration between surgery date and final follow‐up or cancer‐specific death date.

### 3.2 Establishment of prognosis prediction nomogram

**FIGURE 2:**
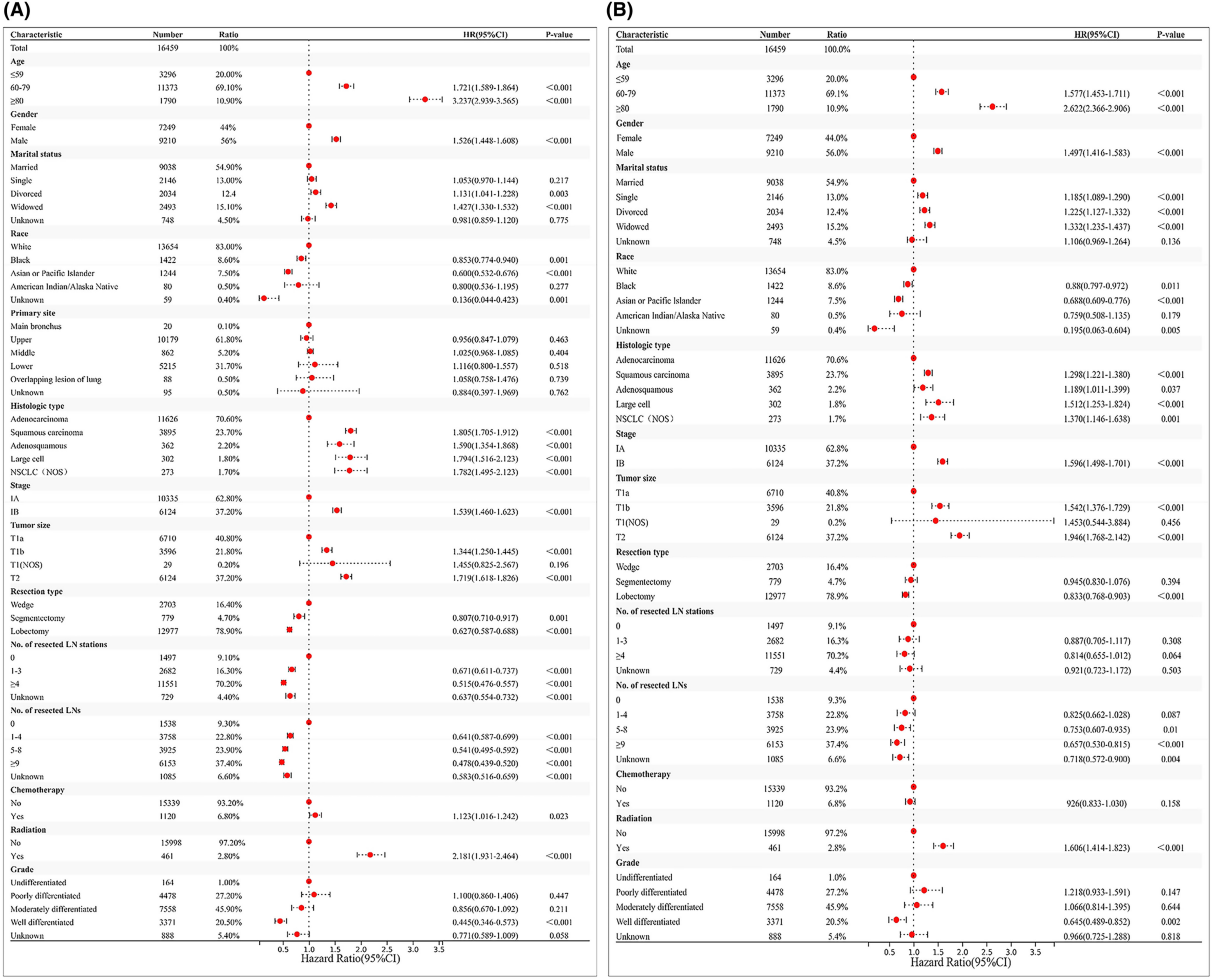
Independent predictors screening. Univariable (A) and multivariable (B) Cox regression of clinicopathological characteristics associated with OS in the SEER Database.

**FIGURE 3:**
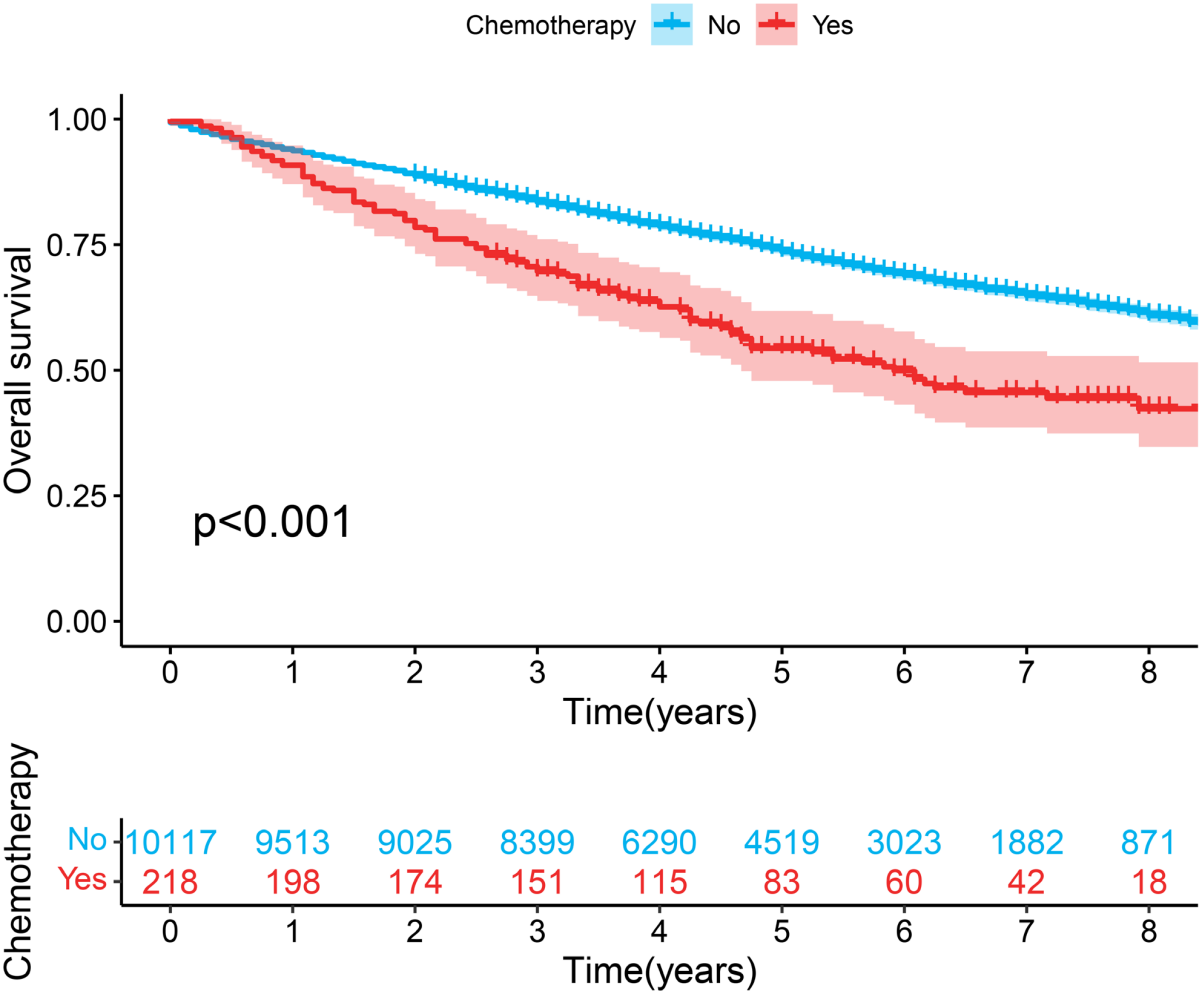
THE Kaplan–Meier survival estimates by chemotherapy, in IA.

**FIGURE 4:**
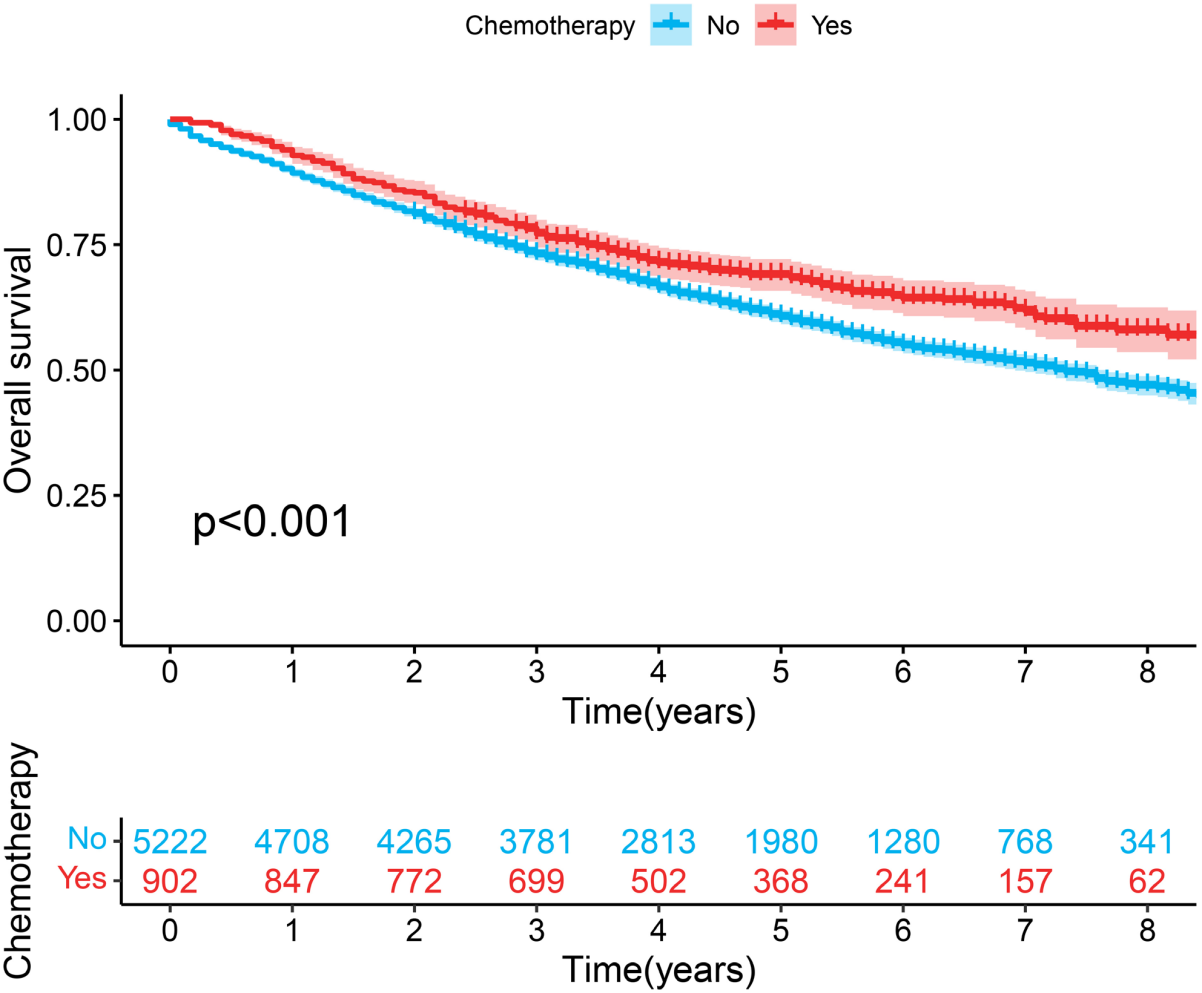
The Kaplan–Meier survival estimates by chemotherapy, in IB.

**FIGURE 5:**
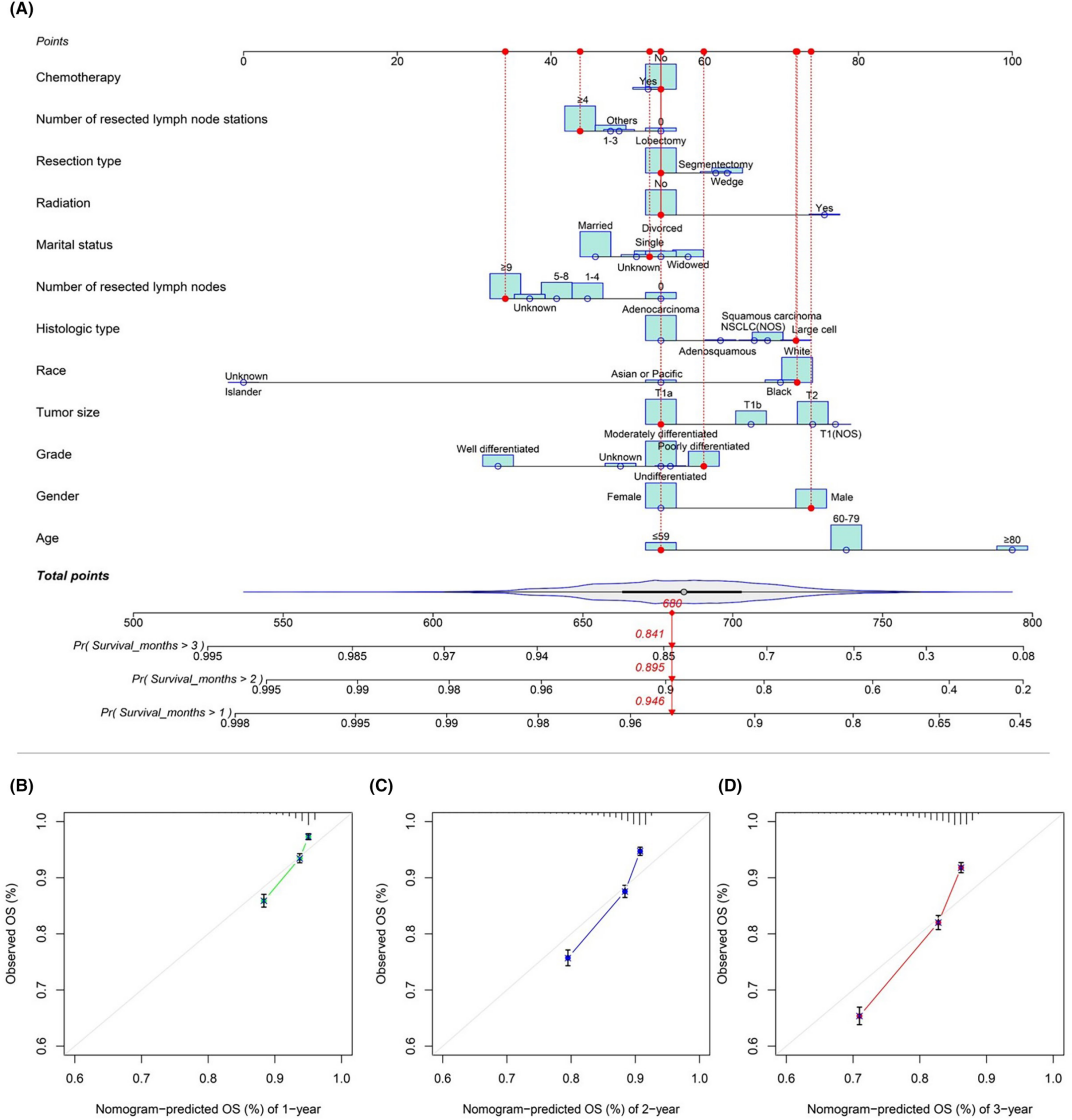
Nomogram construction and validation. (A) A nomogram plot was constructed on the basis of chemotherapy, number of resected lymph node stations, resection type, radiation, marital status, number of resected lymph nodes, histologic type, race, tumor size, grade, gender and age. According to calibration curves, the nomogram‐predicted 1‐ (B), 2‐ (C), and 3‐year (D) OS was well consistent with real OS.

Upon univariable and multivariable Cox regression on SEER cohort of NSCLC patients, Age, Gender, Marital status, Race, Histologic Type, Stage, Tumor size, Resection Type, Resected lymph node station number (No. of resected LN stations), Resected lymph node number (No. of resected LNs), Radiation, Chemotherapy and Grade showed close relation to OS of patients (*p* < 0.05, Figure 2A,B). Chemotherapy was related to a good prognostic outcome of Stage IB NSCLC patients, whereas it was related to the dismal prognostic outcome of Stage IA NSCLC patients (*p* < 0.05, Figures 3 and 4). Based on the multivariate Cox regression result and clinical experience, we developed the clinical prognostic nomogram for predicting patients' 1‐, 2‐, and 3‐year OS probabilities (Figure 5A). There is a clear relationship between the variable location in the nomogram and the contribution to OS. According to variable contribution to survival probability, all variables had their own points (range, 0–100). Using functional transformation relation between the point sum of the various variables and the probability of survival, the total points could be obtained, and separate survival outcomes were predicted by relation of total points with survival probability. We created a web page where a patient's survival risk can be calculated by simply selecting the corresponding clinical factors. The web page link is as follows: https://tongz0225.shinyapps.io/Lung_nomogram/. For instance, one 50‐year‐old (55 points) white (70 points) male (75 points) patient without chemotherapy (55 point) NSCLC after the sum‐point equaled to 680, which indicated the 1‐, 2‐, and 3‐year OS possibilities of 94.6%, 89.5%, and 84.1% respectively.

### 3.3 Evaluation and validation of nomogram

As part of the validation process, the model accuracy and discrimination performance were evaluated by calibration curve, and area under the receiver operating characteristic curve (AUC) analysis for training and validation cohorts. For evaluation of model calibration, we plotted calibration curves. As a result, the predicted survival was well consistent with real survival of training cohort (Figure 5B–D). As shown in the calibration curves, the gray line indicates the reference line, and the predicted survival is consistent with real survival.

**FIGURE 6:**
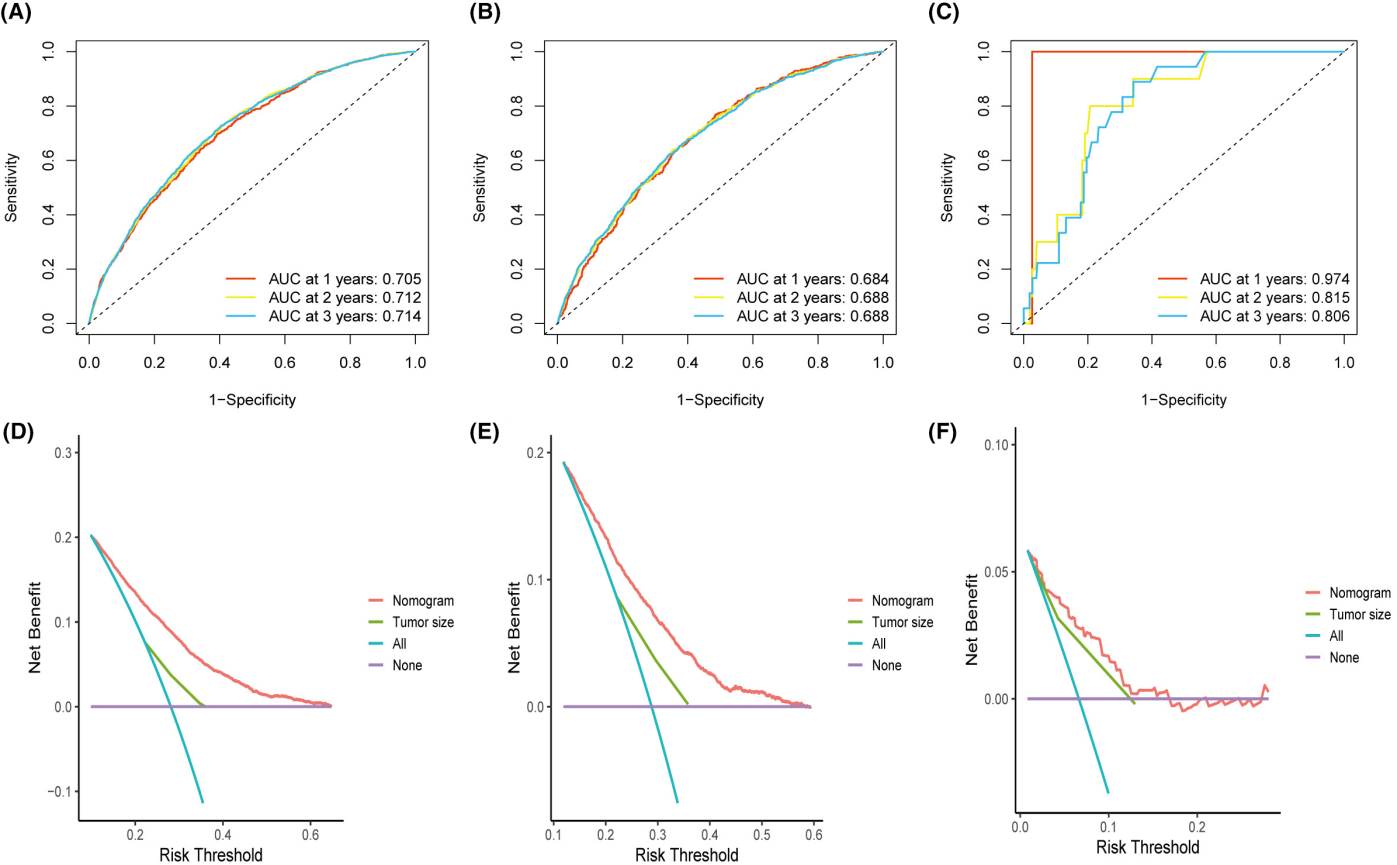
ROC curves of 1‐, 2‐, and 3‐year OS for patients and DCA curves based on nomogram. (A, D) training, (B, E) test, and **(**C, F) external validation cohorts.

In training cohort, AUCs of the nomograms were 0.705, 0.712, and 0.714 for 1, 2, and 3‐year OS separately (Figure 6A). Similarly, those were 0.684, 0.688, and 0.688, separately in test cohort (Figure 6B). Additionally, we utilize external datasets for the validation of our results. As well, we found that data from external datasets showed better results when compared to data from seer datasets (AUCs were 0.974, 0.815 and 0.806 for 1‐, 2‐, and 3‐year OS separately, Figure 6C). Similarly, DCA demonstrated that the nomograms provided superior net benefit to SEER stage, validating that our nomograms were superior to SEER stage (Figure 6D,E). Finally, the C‐index revealed good prognostic accuracy of nomogram with values of 0.684 (95% CI 0.676–0.693) in the training cohort, 0.660 (0.647–0.671) in the test cohort and 0.778 (95% CI 0.708–0.849) in the external validation cohort.

### 3.4 Survival Analyses

**FIGURE 7:**
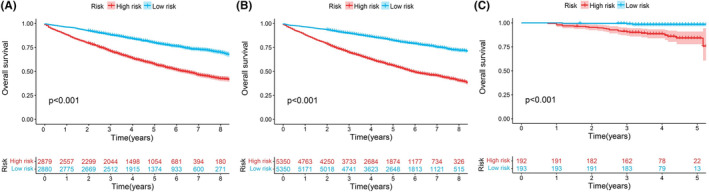
Kaplan–Meier survival curve show overall survival of early‐stage NSCLC patients in different groups. (A‐C) Training cohort (A); test cohort (B); and external validation cohort (C).

The total score of patients was calculated according to the nomogram. We designated patients with nomogram scores of less than the median nomogram score (684 points) as the low‐risk group, and patients with nomogram scores higher than median were identified into a high‐risk group. Both the training and validation sets showed significant differences between the survival curves for both risk groups (*p* < 0.001), conforming to observations of external validation set. High‐risk group showed markedly poorer OS relative to low‐risk group for training and internal validation cohorts (*p* < 0.001, Figure 7). Besides, low‐risk group showed superior OS of external cohort (*p* < 0.001).

## 4 DISCUSSION

We conducted multivariate data suggesting that, as expected, tumor size was positively correlated with the risk of poor differentiation. The mortality rate of Stage I patients is higher in older age, male patients.^24^, ^25^ The risk of white race is higher, followed by Black race, Asians and Indians, which is consistent with previous studies.^25^, ^26^ Chemotherapy is negatively related to prognostic outcome of Stage IA NSCLC patients, whereas positively correlated with that of Stage IB NSCLC patients, conforming to prior reports.^27^ Postoperative radiotherapy can't reduce the mortality in I stage NSCLC patients, but increase patient mortality, which is consistent with previous reports.^28^, ^29^

Lymph node dissection can reduce the postoperative mortality of patients, which may be related to the removal of some potential micrometastatic lesions with more lymph node dissection, and more lymph node dissection can make N staging more accurate.^30^ It is controversial about the lymph node dissected number and extent during the operation. In a previous study, it is reported that in patients with local resection of lung cancer, N1 + N2 resection has a better prognosis than N1 resection.^31^ Our results showed that patients with resected more than 4 lymph stations had a better prognosis. But it is also reported that the number of lymph node station dissection greater than 3 may be a risk factor for postoperative lung cancer.^32^ It has been suggested that removing less than 10 lymph nodes may increase the risk.^25^ Moreover, some studies have found that the optimal range for removal is between 10–11 and 8–14 nodes.^30^, ^33^ Similarly, other studies have reported that the more lymph nodes removed, the better,^34^ which is consistent with our research findings.

Finally, there is a debate about whether the surgical method of early lung cancer is local resection or lobectomy. Studies have shown that there is no difference in morbidity and mortality between the two groups during the perioperative period.^35^ Some previous reports have reported that patients undergoing lobectomy had an increased OS rate compared with those receiving sublobectomy,^36^, ^37^ and other studies have shown that the prognosis of sublobectomy is consistent with that of lobectomy when patients can choose both surgical methods but not for other reasons.^14^ A phase 3 trial shows that sublobar resection is noninferior to lobar resection of IA NSCLC patients when the tumor size is <2 cm.^38^ Therefore, we believe that OS rate of lobectomy may increase relative to sublobectomy, but there may be no difference in the prognosis of those patients who can accept both procedures.

In this nomogram, we included age, sex, race, marital status, pathology, tumor size, resected lymph node number, number of lymph node stations resected, radiotherapy, chemotherapy and grade. These factors are associated with prognostic outcome of lung cancer. Through the SEER database, we use a lot of data for modeling, and the risk will be scored accordingly, demonstrating that prognosis were significantly different in low‐risk compared with high‐risk groups.

Certain limitations should be noted in this work. First, because it is a retrospective study, the data may be biased and inaccurate. Second, due to the limitation of SEER database, the population of different races is biased, and the population of SEER data can't represent all populations. Third, such as the use of postoperative targeted drugs or immune drugs, chemotherapy regimens and doses, whether patients receive neoadjuvant therapy. Finally, the number of samples in our external cohort is small, and only Asians are included and radiotherapy information is not included.

We screened some risk factors associated with postoperative prognosis of lung cancer. Using nomogram, we established a high‐performance tool to predict postoperative prognostic outcome in Stage I patients. In addition, we stratified the risk score of the postoperative population, providing an easy‐to‐use postoperative management prediction tool for doctors and patients to promote individualized postoperative care.

## AUTHOR CONTRIBUTIONS

**Hao Zhang:** Conceptualization (equal); data curation (equal); investigation (equal); software (equal). **Jingtong Zeng:** Conceptualization (equal); data curation (equal); software (equal); writing – original draft (equal). **Xianjie Li:** Data curation (equal); resources (equal); visualization (equal); writing – original draft (equal). **Bo Zhang:** Conceptualization (equal); data curation (equal); visualization (equal). **Hanqing Wang:** Data curation (equal); investigation (equal); visualization (equal). **Quanying Tang:** Conceptualization (equal); formal analysis (equal); visualization (equal). **Yifan Zhang:** Conceptualization (equal); data curation (equal); visualization (equal). **shihao Bao:** Conceptualization (equal); investigation (equal); visualization (equal). **Lingling Zu:** Methodology (equal); supervision (equal); visualization (equal). **Xiaohong Xu:** Supervision (equal); validation (equal); visualization (equal). **Song XU:** Project administration (equal); supervision (equal); writing – review and editing (equal). **Zuoqing Song:** Funding acquisition (equal); project administration (equal); writing – review and editing (equal).

## FUNDING INFORMATION

The present study was funded by the National Natural Science Foundation of China (82172776), Tianjin Science and Technology Plan Project (19ZXDBSY00060), Tianjin Key Medical Discipline (Specialty) Construction Project (TJYXZDXK‐061B), and Diversified Input Project of Tianjin National Natural Science Foundation (21JCYBJC01770).

## CONFLICT OF INTEREST STATEMENT

The authors declare that the research was conducted in the absence of any commercial or financial relationship.

## ETHICS STATEMENT

All tissue specimens were used after approval from the Ethics Committee of Tianjin Medical University General Hospital (Ethical No. IRB2021‐WZ‐055). The patients/participants provided their written informed consent to participate in this study. Written informed consent was obtained from the individual(s) for the publication of any potentially identifiable images or data included in this article.

## Supporting information


Data S1:

## Data Availability

The SEER and NCDB datasets are available upon request from the NCI and ACS. Coding scripts are available from the authors upon request.
